# A Novel Phase Variation Mechanism in the Meningococcus Driven by a Ligand-Responsive Repressor and Differential Spacing of Distal Promoter Elements

**DOI:** 10.1371/journal.ppat.1000710

**Published:** 2009-12-24

**Authors:** Matteo M. E. Metruccio, Eva Pigozzi, Davide Roncarati, Francesco Berlanda Scorza, Nathalie Norais, Stuart A. Hill, Vincenzo Scarlato, Isabel Delany

**Affiliations:** 1 Novartis Vaccines and Diagnostics, Siena, Italy; 2 Department of Biology, University of Bologna, Bologna, Italy; 3 Department of Biological Sciences, Northern Illinois University, DeKalb, Illinois, United States of America; Northwestern University Feinberg School of Medicine, United States of America

## Abstract

Phase variable expression, mediated by high frequency reversible changes in the length of simple sequence repeats, facilitates adaptation of bacterial populations to changing environments and is frequently important in bacterial virulence. Here we elucidate a novel phase variable mechanism for NadA, an adhesin and invasin of *Neisseria meningitidis*. The NadR repressor protein binds to operators flanking the phase variable tract and contributes to the differential expression levels of phase variant promoters with different numbers of repeats likely due to different spacing between operators. We show that IHF binds between these operators, and may permit looping of the promoter, allowing interaction of NadR at operators located distally or overlapping the promoter. The 4-hydroxyphenylacetic acid, a metabolite of aromatic amino acid catabolism that is secreted in saliva, induces NadA expression by inhibiting the DNA binding activity of the repressor. When induced, only minor differences are evident between NadR-independent transcription levels of promoter phase variants and are likely due to differential RNA polymerase contacts leading to altered promoter activity. Our results suggest that NadA expression is under both stochastic and tight environmental-sensing regulatory control, both mediated by the NadR repressor, and may be induced during colonization of the oropharynx where it plays a major role in the successful adhesion and invasion of the mucosa. Hence, simple sequence repeats in promoter regions may be a strategy used by host-adapted bacterial pathogens to randomly switch between expression states that may nonetheless still be induced by appropriate niche-specific signals.

## Author Summary

Diversification strategies, through genetic switches that randomly turn genes on and off, occur in many pathogenic bacterial populations and confer adaptive advantages to new environments and evasion of host immune responses. This is often mediated by spontaneous changes in the length of short DNA sequence repeats located in protein-coding regions or upstream regulatory regions, leading to deactivation or alteration of the associated genes. In this study we describe how a repeat sequence, distally upstream of the promoter region, alters the expression of an important adhesin of *N. meningitidis*. We identify the major mediator of this control, a negative regulator NadR, which binds to sequences flanking the variable repeat. Changes in the spacing between these sequences affect the ability of NadR to shut down expression from the promoter. We also identify a relevant metabolite that can block NadR activity and therefore act as a signal to induce adhesin expression. This finding sheds new light on the role of DNA-repeats identified in intergenic regions for which no role could be hypothesised, and may be a model mechanism used by bacterial pathogens for fine-tuning diversity within the host. Elucidating these mechanisms can aid in our understanding and prevention of disease.

## Introduction

*Neisseria meningitidis* is an important human pathogen which colonises the nasopharynx in about 5–10% of healthy individuals. Occasionally, and for reasons not fully understood, it can cause an invasive infection leading to septicaemia and also meningitis [1],[2]. In these cases, the meningococcus can rapidly undergo transcytosis across the epithelial and endothelial barriers into the bloodstream, where efficient replication and dissemination occurs. Consequently, the organism is able to cross the blood/brain barrier gaining access to the meninges surrounding the brain as well as infecting other organs. In order to ensure effective colonization and transmission, as well as coping with the diverse stages of the infectious cycle inside the host, the meningococcus must be able to respond and adapt to different microenvironments through regulated and stochastic expression of genes involved in pathogenesis. The *nadA* gene, coding for an adhesin and invasin of meninogococcus [3],[4] is an important gene involved in bacterial pathogenesis, whose gene product is one of the components of a potential vaccine against meningococcal serogroup B outbreaks [5],[6].

The *nadA* gene is known to be present in approximately 50% of meningococcal isolates and is absent in *N. gonorrhoeae* and in commensal *Neisseriae* [3]. Due to the low %GC content of the *nadA* locus, it is thought to have been acquired in the meningococcus by horizontal transfer. NadA expression was shown to exhibit growth-phase dependent behaviour with levels reported to be maximal in the stationary growth phase of all strains tested [3]. Furthermore, the expression of NadA is phase variable and a tetranucleotide tract (TAAA) upstream of the *nadA* promoter has been demonstrated to control this phenomenon [7]. In *Neisseria*, phase variation of many genes is associated with reversible changes within simple DNA sequence repeats located in coding or promoter regions of genes [8]. The number of repeats can be modified during replication through slipped strand mispairing [9], and can consequently influence translation or transcription by introducing frameshift mutations or changing critical promoter spacing [10],[11],[12],[13]. The loss or gain of repeat units results in high frequency on-off switching (in the case of frameshift/translational control) or modulation of the level (in the case of promoter control) of expression of genes usually associated with surface-exposed antigens.

The phase variable tract of *nadA* is unique, as it is distally located upstream of the *nadA* promoter, unlike the phase variable repeat tracts found in the *porA, fetA*, and *opc* genes where the unstable homopolymeric stretches are found between the −10 and the −35 promoter elements and are thought to result in altered sigma-factor binding [10],[14],[15]. The frequency of phase variation of *nadA* has been experimentally estimated as ca. 4.4×10^−4^ [7] creating variants where changes in the repeat number result in promoters with low, medium or high activity. The transcriptional regulators Fur and IHF were implicated in the control of *nadA* promoter activity from the binding of both proteins to the *nadA* promoter and from the analysis of mutants deleted for IHF- and Fur-binding sites [16]. Moreover, it has been reported that loss or gain of a tetranucleotide repeat affects the binding of the IHF regulatory protein to the *nadA* promoter in vitro, and this was proposed to be responsible for the modulation of transcription of *nadA in vivo* [16]. Nonetheless, the mechanism governing transcriptional regulation of *nadA* remains unclear and the inferred role of IHF or Fur and their involvement in phase variation of *nadA* expression remain to be elucidated. However, a novel regulator of NadA expression has recently been identified which was shown to repress NadA expression [17].

In this study we dissect the cis- and trans-acting elements involved in transcriptional regulation of *nadA* as well as describe an environmental factor that appears to induce expression of the NadA protein. We propose a novel mechanism by which the spontaneous changes in the number of simple sequence repeats distally located with respect to the core promoter can alter the promoter activity and lead to phase variable expression.

## Results

### All phase variant promoters are growth-phase regulated

**Figure 1:**
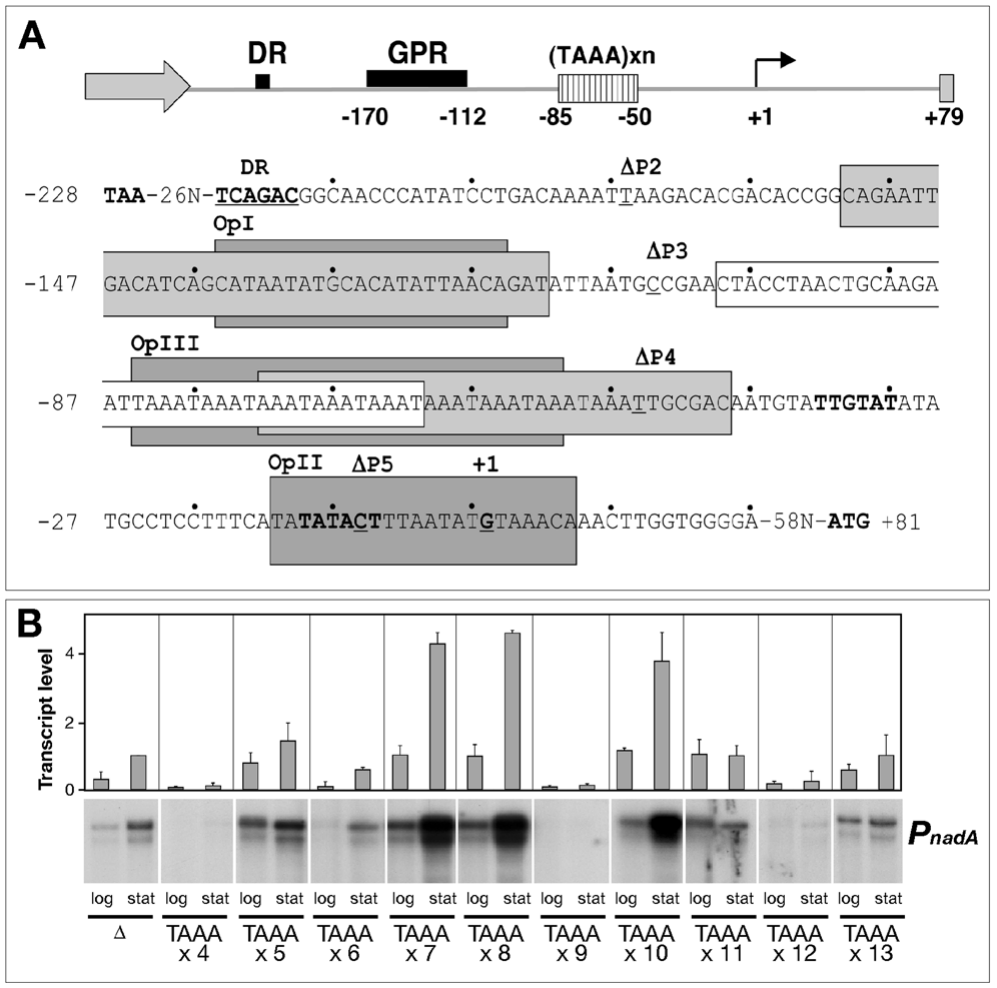
The P*_nadA_* promoter and transcript level. (A) Schematic diagram of the P*_nadA_* elements. DR, direct repeat (border of region of horizontal transfer); GPR, growth phase regulatory region; ΔP2-ΔP5 indicates the nucleotide positions of the 5′ deletion mutants (Figure 3). The nucleotide sequence of the promoter is shown with the regions bound and protected in DNase I footprinting shaded according to the regulatory proteins tested in vitro: light grey, RNAP α-subunit; white, IHF; dark grey, NadR. (B) Transcription of each phase variant promoter is growth phase responsive. Cultures of MC-PΔ, MC-P2(x4), MC-P2(x5), MC-P2(x6), MC-P2(x7), MC-P2(x8), MC-P2(x9), MC-P2(x10), MC-P2(x11), MC-P2(x12), MC-P2(x13) strains, carrying single copy transcriptional fusions of the phase variant *nadA* promoter with a defined number of copies of the tetranucleotide repeat (TAAAxN) and the repeated tract deleted (Δ) (Table 1), were grown to mid-log or stationary growth phase and total RNA was prepared. Quantitative primer extension was performed using a *gfp*-specific primer as described in materials and methods. Autoradiographs of a representative experiment are shown as well as the quantification of transcript levels as determined by phosphorimaging. The error bars on the graph represent the standard deviations observed for the quantification of transcript levels between at least 2 biological replicates.

Previous analysis of NadA expression in several meningococcal isolates indicated that its expression is controlled by variation in the number of tetranucleotide repeats (TAAA) upstream of the core promoter [7] and that the protein is maximally expressed in stationary growth phase [3]. In order to study transcriptional regulation of the *nadA* promoter we generated isogenic *N. meningitidis* MC58 strains, each carrying a *nadA* phase variant promoter fusion with a defined number of repeats and determined the relative level of the *nadA* transcripts. Steady state levels of *nadA-gfp* transcript were measured by quantitative primer extension analyses on RNA extracted from cells grown to the mid log and the stationary growth phases. Figure 1 shows key elements of the *nadA* promoter (panel A) and demonstrates the varying pattern of transcript level between promoters with different numbers of TAAA repeats (panel B). As previously reported [16], we confirm that 4, 9 and 12 repeats result in low transcript level, and show that 7, 8 and 10 repeats result in high transcript level, and 5, 6, 11, 13 repeats and a promoter mutant lacking TAAA repeats (Δ) give varying intermediate levels, which when taken together represent a quasi-periodic pattern in the transcript level. Furthermore, each phase variant promoter exhibits a certain degree of growth-phase dependent transcription, with a higher level of transcription in stationary growth phase.

### Regulatory proteins that bind the nadA promoter

In vitro DNA binding assays suggested that regulation of *nadA* expression is under the control of the Fur and IHF regulatory proteins and that loss or gain of TAAA repeats could affect IHF binding, thus accounting for the different promoter activity of the phase variants [16]. In order to gain insight into the molecular mechanism controlling *nadA* expression, we mapped the precise location where Fur, IHF, and RNA polymerase (RNAP) bind to the *nadA* promoter. DNase I footprinting was performed with the purified proteins and three radioactively labelled phase variant promoters, corresponding to low (9 repeats), medium (6 repeats), and high (7 repeats) transcript level.

Addition of increasing amounts of a recombinant Fur protein (0.013–3.2 µM) showed a region of protection at 3.2 µM Fur concentration (data not shown). This protection overlapped the translational start site (+79) spanning from +61 to +96. However, no differences in *nadA* transcription were detected in a Fur null mutant background when compared with the wild type strain, or in response to changing iron concentrations (data not shown). Therefore, the observed in vitro binding of Fur to the *nadA* promoter appears to have no correlation with in vivo regulation of transcription by Fur in response to iron.

**Figure 2:**
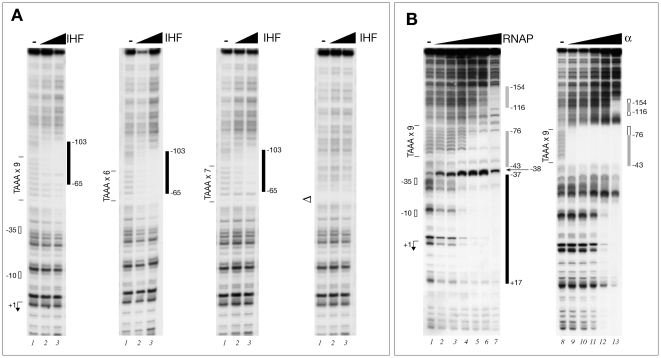
Regulatory proteins binding to the *nadA* promoter. (A) DNase I footprinting of IHF protein to three different phase variant *nadA* promoters with 9, 6 and 7 repeats corresponding to low, medium and high transcript level in vivo, respectively, and the PΔ mutant *nadA* variant with a deletion of the TAAA repeated tract. To 20 fmoles of each radioactively labelled probe, 0, 43 and 172 nM (lanes 1–3) of IHF heterodimer were added. Relevant regions are marked and numbers correspond to nucleotide positions with respect to the transcriptional start site of a promoter with 9 repeats. (B) DNase I footprinting of RNAP or the α-subunit of RNAP to the indicated *nadA* promoter probe. The probe was incubated with 0, 0.25, 0.5, 1, 2, 4, and 5 U of RNAP (lanes 1–7) or 0, 0.17, 0.68, 2.7, 5.5, 11 µM of purified α-subunit (lanes 8–13).

Addition of 43 or 172 nM of the IHF heterodimer to the binding reactions resulted in a similar region of protection in all three phase variant probes (Figure 2A). IHF binds upstream of the distal border of the TAAA tract and the protection spans the first 5 repeats, from −103 to −65 with respect to the promoter with 9 repeats (Figure 2A). Accordingly, no binding could be detected in a similar footprinting experiment with the PΔ promoter variant in which the TAAA tract was deleted (Figure 2A). Notably, variations of the number of repeats from 6 to 9 resulted in no differential binding of IHF.

As expected, addition of RNAP to the *nadA* promoter probe resulted in a characteristic footprint over the core promoter spanning from −37 to +17, as well as protecting two other regions, one directly upstream of the core promoter spanning positions −43 to −76, partially overlapping the TAAA tract, and the second distally upstream spanning from −116 to −154 (Figure 2B). As both upstream protected regions are AT-rich regions, a typical feature of UP-like elements bound by the C-terminal region of the α-subunit of RNAP to enhance transcription [18],[19], we decided to verify such a hypothesis in vitro by DNase I footprinting using the purified α-subunit of the RNAP. Results showed a specific binding of the α protein over the TAAA repeats at low protein concentration (Figure 2B). Upon addition of increasing amounts of the α protein, this protected region extended both to downstream and upstream regions, including regions spanning positions −43 to −76 and −116 to −154 protected by the holoenzyme (Figure 2B). Furthermore, because the *nadA* promoter is recognised and transcribed from the same +1 in *E. coli* (data not shown), we decided to test whether the α-subunit of RNAP could play a role in the transcription of P*_nadA_* in this system. We measured promoter activity of a P*_nadA_-gfp* fusion (on plasmid pGX-nad-gfp) in an *E. coli* strain over-expressing either a wild type α-subunit (RpoA) or a C-terminally truncated α–subunit (RpoAΔ256) of *E. coli*. Expression of the P*_nadA_-gfp* fusion in the strain over-expressing the wild type α-subunit gave 6393±254 Units (fluorescence normalized with OD_600_), while in the strain over-expressing the α truncated version the activity was reduced by over 50% giving 2867±63 Units. No reduction in promoter activity was apparent when the PΔ *nadA* fusion was co-expressed with the α or truncated α subunit (data not shown). These data suggest that the incorporation of a complete α-subunit into the RNAP allows maximum transcriptional activity at P*_nadA_*, possibly through contacts of the C-terminal region of the α-subunit to upstream DNA regions containing AT-rich sequences sharing similarities to an UP element.

In conclusion, we have mapped multiple specific points of contact for regulatory proteins on the *nadA* promoter, including distal and proximal binding sites for the α-subunit of RNAP which flank a single IHF binding site at the distal junction of the TAAA tract. IHF is well known for its ability to bend DNA by up to 180° [20], and this property may permit looping of the DNA and the interaction of regulators at distal operators and the transcriptional machinery over the promoter.

### Identification of a cis-acting growth phase regulatory (GPR) region in P_nadA_ and the protein that binds to it

**Figure 3:**
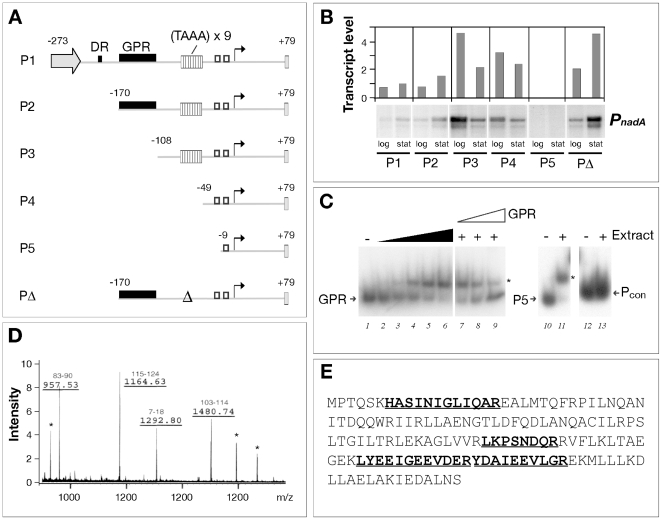
Identification of a *cis*-acting element of the *nadA* promoter determining growth phase regulatory effects (the GPR region) and the GPR-binding protein from cleared cell extracts of MC58. (A) Schematic representation of the mutant *nadA* promoter variants (based on the MC58 *nadA* promoter with 9 repeats) present in single copy transcriptional fusion in the MC58 background in the strains, MC-P1, MC-P2, MC-P3, MC-P4, MC-P5, MC-PΔ. The numbers indicate nucleotide positions with respect to the +1 transcriptional start site. DR, direct repeat; GPR, growth-phase regulatory region; Δ, deletion of the TAAA repeats. (B) Transcription from the mutant promoter variant fusions in log and stationary phases. The MC-P1, MC-P2, MC-P3, MC-P4, MC-P5, MC-PΔ strains were grown to mid-log and mid-stationary growth phase and total RNA was prepared from each sample. Quantitative primer extension was performed as described in materials and methods. Autoradiographs of a representative experiment are shown as well as the quantification of transcript levels. Similar results were found for deletion variants carrying 11 TAAA repeats (data not shown) although the overall transcript levels for promoters containing 11 repeats was higher than that of 9 repeats, as expected. The relative quantities between biological replicates with different numbers of repeats were reproducible within an error of 20% of the absolute value for each mutant promoter. (C) Binding activity towards the *nadA* promoter in cell extracts of MC58. Cell extracts were prepared from mid-log cultures of MC58 and increasing quantities were incubated with a radioactively labelled DNA probe consisting of the GPR region (−170 to −108) or P5 (−9 to +79) or an unrelated intergenic region Pcon as negative control and submitted to EMSA analysis. To ca. 80 fmoles of radioactively labelled probe, 0, 0.2, 0.6, 1.8, 5.0, 15 µg of cell extract in lanes 1–6 were added, respectively; 0 µg in lanes 10 and 12; and 15 µg in lanes 7–9, 11 and 13, were added; and 130, 400, and 1000 fmoles of cold GPR probe in lanes 7, 8, and 9 were added as specific competitor. (D) The peptide mass fingerprint spectrum of one µl of the eluted fraction after DNA affinity purification of the binding factor of the GPR region. Four of the major ions, labelled, could be assigned to tryptic peptides (positioning of the amino acids indicated above) of the NadR transcriptional regulator protein. In addition, BSA was added during the process of purification, was eluted from the column, since 3 major signals observed in the spectrum corresponded to BSA tryptic peptides (marked with an asterisk). (E) The amino acid sequence of the NMB1843 (NadR) protein showing the peptides that were identified by MS in bold and underlined.

In order to identify regulatory regions within the P*_nadA_* promoter we created a range of deletion mutants and measured the transcript level from cells grown to the mid-log and stationary growth phases (Figure 3A and 3B). While deletion of nucleotide sequences upstream of −170 with respect to the +1 transcriptional start site had little or no effect on the level of transcript (promoter P2 versus P1), promoter mutants lacking the region between −170 and −108, (P3 or P4) resulted in a significant increase in transcription during log phase. This finding indicates that the growth-phase dependent regulation is due to a repression of expression in log phase. Accordingly, removal of the TAAA tract did not alter the growth-phase regulation of the resultant mutants (P4 versus P3, or PΔ versus P2). Therefore, we have identified a distal upstream cis-acting region that we call the GPR region, which is responsible for repression of transcription from P*_nadA_* in log phase, possibly upon binding of a repressor protein.

To assess that a repressor factor could bind the GPR region we analysed crude cell extracts of the MC58 strain for the ability to retard a radioactively labelled GPR probe in Electrophoretic Mobility Shift Assays (EMSA). Addition of 15 µg of MC58 extracts resulted in a shift of the GPR probe, which could be outcompeted with cold GPR DNA but not with non-specific competitor (Figure 3C). We also found that the P5 promoter probe spanning from −9 to +79 of the P*_nadA_* promoter was specifically retarded (lane 11, Figure 3C) by MC58 extracts but not an unrelated intergenic region (Pcon) used as negative control (lane 13, Figure 3C). Subsequently, to identify this factor we performed DNA affinity purification using the biotinylated GPR region as ‘bait’. The bound material was digested with trypsin, and the resulting peptides were analyzed by MALDI-TOF mass spectrometry. Four of the seven major ions could be assigned to tryptic peptides derived from the NMB1843 protein (Figure 3D and 3E). To confirm the interpretation, the major parental ions were fragmented. Spectra of fragmentation were consistent with the expected NMB1843 amino acid sequence (data not shown). We call this protein that binds the GPR region of the *nadA* promoter NadR. The *nadR* gene encodes a transcriptional regulator of the MarR family of repressors, is a homologue of FarR, the repressor of the fatty acid resistance efflux pump of *N. gonorrhoeae* [21],[22], and was recently implicated as a repressor of *nadA* [17]. We rename the meningococcal homologue NadR as, unlike the FarR protein, it does not regulate the fatty acid efflux pump in the meningococcus (Pigozzi E, unpublished data) and, therefore, is not involved in fatty acid resistance.

### NadR binds to three operators in the nadA promoter

**Figure 4:**
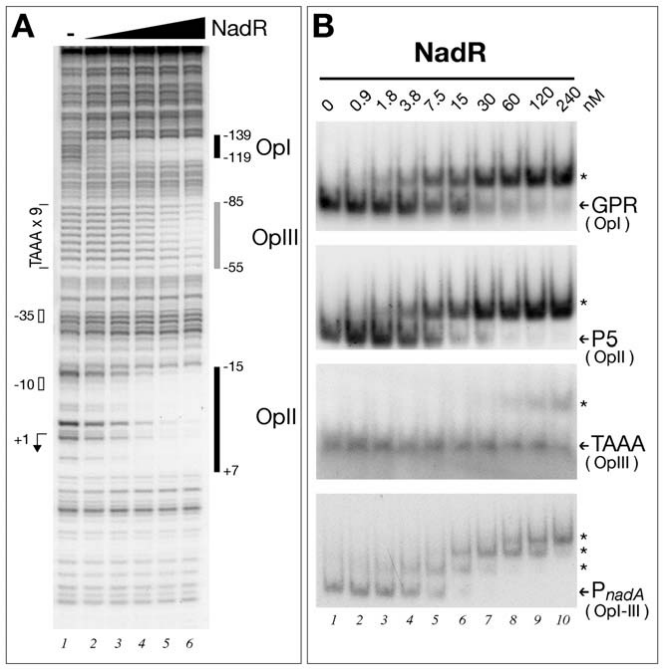
The NadR repressor binds specifically to three operators in the *nadA* promoter. (A) DNase I footprinting analysis with purified NadR on the *nadA* promoter with 9 repeats. The NadR protected regions are indicated (OpI–III) and numbers represent the nucleotide positions with respect to the transcriptional start site. The size of protected regions ranges from 20 bp (OpI and OpII), and 30 bp (OpIII), a size compatible with the binding of a protein dimer. Furthermore, in vitro cross-linking experiments with the purified NadR protein revealed the presence of cross-linked oligomers which migrated on SDS-PAGE with a molecular weight compatible with a dimer (data not shown). Therefore, NadR, similarly to other members of the MarR family of proteins is likely to be a dimer in solution. Binding reactions contained 40 fmoles of probe radioactively labelled at one extremity and 0, 7.5, 15, 30, 60, 120 nM of NadR purified dimer (lanes 1–6, respectively). (B) EMSA with radioactively labelled GPR, TAAA and P5 probes containing the individual OpI, OpIII and OpII operators, respectively, or the entire P2 *nadA* promoter spanning from −170 to +79 with increasing concentrations of recombinant NadR protein as indicated. The retarded migration of protein DNA complexes are indicated with asterisks.

To demonstrate that NadR is the GPR-binding factor we generated a deletion *nadR* mutant by substituting the gene with an antibiotic resistance marker. Cell extracts derived from the *N. meningitidis* Δ1843 mutant no longer possessed binding activity towards the GPR and P5 promoter probes (data not shown). We amplified and cloned the *nadR* gene from the MC58 genome into an expression plasmid and expressed and purified a recombinant form of the protein with an N-terminal Histidine tag. We performed DNase I footprinting analysis with the NadR protein and a radioactively labelled probe consisting of the entire *nadA* promoter. Figure 4A shows the autoradiogram of the results. On addition of increasing amounts of NadR recombinant protein, three regions of protection of the *nadA* promoter are visible. Two appear on addition of 30 nM of NadR protein: the first (OpI) spanning from −139 to −119 and the second (OpII) spanning from −15 to +7 and, therefore, within regions of the GPR and P5 probes that were previously shown to be bound by the MC58 extracts as well as a third region (OpIII) spanning the TAAA tract from −55 to −85. EMSA analysis confirmed that NadR exhibits high affinity for the GPR and P5 operator regions and exhibits a lower affinity for the TAAA tract. These observations were supported by EMSA analysis with a probe spanning the entire P*_nadA_* promoter as three differential protein-DNA complexes were formed, most likely following sequential binding of the protein to the operators located within the P*_nadA_* probe (Figure 4B).

From this analysis we conclude that NadR encodes the GPR-binding repressor factor that binds to three operators; two high affinity operators OpI and OpII within the distal GPR region and overlapping the *nadA* promoter, respectively, and a lower affinity operator OpIII which spans the TAAA repeat tract.

### NadR represses differentially phase variant promoters

**Figure 5:**
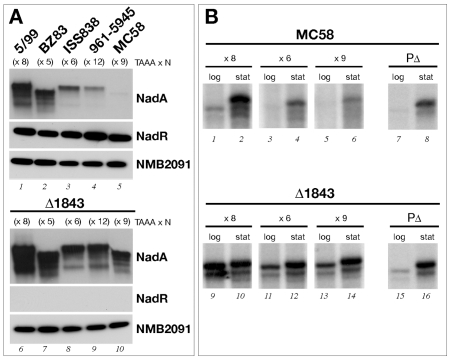
The NadR repressor contributes to phase variable expression. (A) Western Blot analysis of the level of expression of NadA and NadR in wild type strains 5/99, BZ83, ISS838, 961–5945 and MC58 carrying *nadA* promoters with 8, 5, 6, 12, and 9 repeats (lanes 1–5), respectively, and their NadR null mutant derivatives, 5/99- Δ 1843, BZ- Δ 1843, ISS-Δ1843, 961-Δ1843, MC-Δ1843 (lanes 6–10). Cells were recovered from overnight culture on plates and 5 µg of total protein were loaded on SDS-PAGE, blotted and stained with anti-NadA, anti-NadR, or anti-NMB2091 polyclonal antiserum. Migration of the NadA proteins is altered as these strains express NadA proteins with variations in their amino acid sequences [3], however the promoter sequence in each strain is identical apart from the altered number of repeats. (B) Transcription of phase variant promoters with 8, 6, 9, and, no, repeats, in the MC58 and NadR null mutant backgrounds. Total RNA was prepared from cultures of strains MC-P2(x8), MC-P2(x6), MC-P2(x9), MC-PΔ, Δ1843-P2(x8), Δ1843-P2(x6), and Δ1843-P2(x9), Δ1843- PΔ, grown to mid-log and stationary growth phase. Quantitative primer extension was performed as described in materials and methods. A representative experiment is shown. The experiment was performed on at least 2 biological replicates and the standard deviations between the values did not exceed 20% of the value.

To further study the role of NadR in regulating NadA expression, and its possible involvement in mediating differential expression from phase variant promoters, we first selected five representative strains bearing different numbers of tetranucleotide repeat in their *nadA* promoter which correlate to high (8 repeats, 5/99) and low (9 repeats, MC58) as well as three intermediary (5 repeats, BZ83; 6 repeats, ISS838, and 12 repeats, 961–5945) levels of NadA expression and generated isogenic knockouts of NadR in each background. The level of expression of NadA and NadR in the wild type and Δ1843 meningococcal strains was evaluated by Western Blot, in order to evaluate the role that NadR may play in NadA regulation across different strains of the meningococcus. The wild type strains showed, as expected, levels of NadA expression that can be associated with transcript levels of the *nadA* phase variant promoter they bear, and NadR was constitutively expressed in each strain (Figure 5A, lanes 1–5). Each of the knockout strains exhibits higher levels of NadA expression than their respective wildtype strain indicating that NadR represses *nadA* expression in each strain (lanes 6–10 vs 1–5). Surprisingly, the mutation of NadR results in almost equivalent levels of NadA between the knockout strains, although the 5/99-Δ1843 and BZ-Δ1843 still exhibit slightly higher NadA expression. This suggests that NadR, although expressed to the same level, has a different repressive activity on the *nadA* gene in each strain and this may depend on the number of repeats in the different phase variant promoters i.e. NadR does not efficiently repress the 8x promoter of 5/99 but very efficiently represses the 9x promoter of MC58. To further test this hypothesis and to rule out effects due to strain differences, we deleted the *nadR* gene in the isogenic MC58 strains carrying high (x8), medium (x6) and low (x9) promoter variants and measured the steady state levels of transcription from the promoters at log and stationary growth phase in the presence or absence of the NadR regulator. The results in Figure 5B confirm that in the mutant (Δ1843) all three promoters are derepressed, and, interestingly, little or no variation in transcript levels between the phase variants is observed, suggesting that in the absence of NadR the mechanism of transcriptional control exerted by variable number of repeats is alleviated or negligible. It is worth noting, however, that the maximum level of transcription in exponentially growing cells is observed from the promoter variant with 8 repeats, in agreement with higher NadA expression in 5/99-Δ1843, suggesting that NadR is not the sole modulator of phase variable promoter activity and that there is another factor which may establish differential RNAP contacts to modulate transcription.

Furthermore, we also measured the transcript level of the PΔ promoter, which lacks the TAAA tract and also no longer binds IHF, in the wild type and Δ1843 backgrounds and results indicate that NadR does not efficiently repress this mutant promoter (lanes 15 and 16 versus 7 and 8) and implicates a major role for IHF in efficient NadR-mediated repression of the *nadA* promoter.

### Ligand-responsive regulation of NadA expression

**Figure 6:**
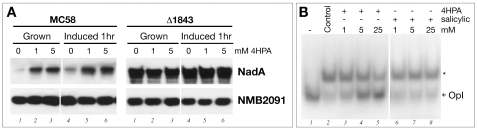
Ligand-responsive regulation of NadA expression. (A) Induction of expression of NadA by addition of a small molecule ligand 4HPA. Broth cultures of MC58 or Δ1843 were grown to OD_600_ of 0.24 without (lane 1) or with 1 mM or 5 mM (lane 2 and 3) 4HPA; or to OD_600_ of 0.24 and then incubated with 0, 1 or 5 mM 4HPA (lanes 4–6) added for 1 h. Cells were harvested and 5 µg of total protein from each culture was subjected to SDS-PAGE and Western Blot analysis with anti-NadA or anti-NMB2091 antibodies as negative control. (B) EMSA assays demonstrating dissociation of NadR from OpI operator in the GPR probe in vitro following the addition of 4HPA (lanes 3–5) but not the broadly acting salicylic acid ligand (lanes 6–8).

The MarR family of proteins regulates a wide variety of biological processes including resistance to antibiotics and antimicrobial agents, virulence and environmental sensing of aromatic compounds [23],[24]. They respond to small inducer molecules which attenuate the ability of MarR homodimers to bind their cognate DNA sequences [23], and are often the molecular substrates for the efflux pumps or metabolic pathways that are repressed by this family of regulators. We set about identifying a small molecule inducer, which may regulate NadR-mediated repression of NadA expression in the meningococcus. We assessed broad-specificity inducers such as salicylic acid, which have been shown to be active against many members of this family, and also functionally relevant molecules such as long-chain fatty acids, which are the substrate for the regulated efflux pump of the gonococcal NadR homologue FarR [22] with no success. However, we noticed that immediately downstream of the *nadR* gene is an ORF which encodes a putative flavoprotein oxidoreductase with 42% amino acid identity to the small subunit of 4-hydroxyphenylacetic acid 3-hydroxylase. In addition, the closest BLAST neighbour of NadR in the MarR family of repressors is the HpaR protein (50% identity), which represses the 4-hydroxyphenylacetic acid (4HPA) catabolic pathway in *E. coli*. Moreover, it is responsive to the 4HPA substrate of the pathway, which binds to the repressor and induces expression of the catabolic genes [25]. We, therefore, assessed whether the 4HPA molecule could act as putative inducer of NadA expression in vivo. Addition of 1 mM or 5 mM 4HPA (Figure 6A) to cultures of wild type MC58 significantly induced NadA expression. No induction could be detected in cultures of the Δ1843 mutant, indicating that the 4HPA molecule induced a NadR-mediated derepression of NadA expression. To confirm that the observed increases in NadA expression could represent a direct interaction of the inducer with NadR, the ability of the compound to dissociate purified recombinant NadR from the high affinity operator OpI was assessed by EMSA. The 4HPA compound was found to attenuate the binding activity of the NadR regulator to the GPR probe in vitro (Figure 6B). Furthermore, addition of 1 mM 4HPA to crude cell extracts containing the native NadR meningococcal protein resulted in complete inhibition of retardation of the GPR probe in EMSA (data not shown), suggesting that the recombinant and native NadR proteins respond in vitro similarly to the compound. These data suggest that the 4HPA could be a ligand of the NadR repressor and interaction of the ligand with the protein attenuates the DNA-binding activity of the molecule for its specific operators and results in derepression or induction in vivo of the *nadA* promoter.

## Discussion

Phase variation is the adaptive process by which bacteria undergo frequent and reversible phenotypic changes resulting from genetic alterations in specific loci of their genomes and this process is crucial for the survival of pathogens and commensals in hostile and ever-changing host environments. *N. meningitidis* has an unprecedented potential for generating genetic diversity through slipped strand mispairing of simple sequence repeats, as its genome contains over 100 repeat associated genes [26],[27]. The way in which genes are affected by variation in the number of repeats is largely thought to occur through biphasic on/off translational control due to frameshifting within the ORFs of coding regions. Recently the on/off switching of methyltransferase genes has been shown to co-ordinate expression of a phase-variable regulon of genes or “phasevarions” via differential methylation of the genome [28],[29]. The role of SSR in intergenic regions in modulating phase variable expression, although frequently found, are less easy to predict. However, differential spacing due to SSR tracts between the core promoter elements modulating multi-phasic expression by affecting RNAP sigma factor binding has been frequently reported [10],[15],[30],[31] as well as some documented examples where repeats in 5′UTR [32],[33] and distally upstream [7],[32],[34],[35] of promoters have been shown to affect expression through unknown mechanisms.

**Figure 7:**
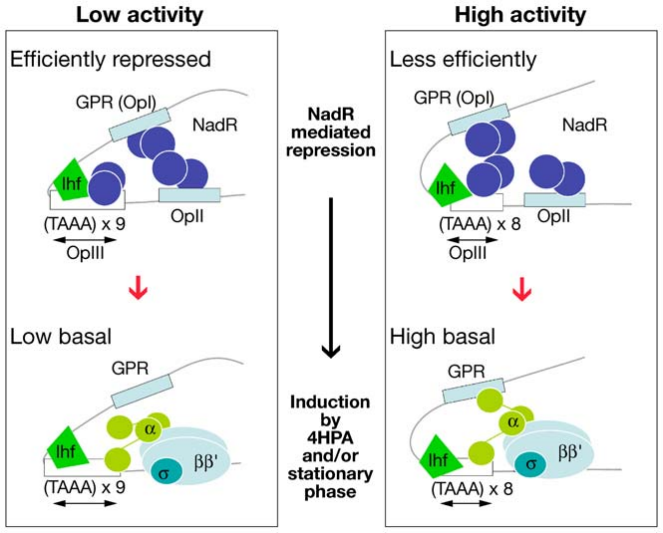
Model of regulation of NadA promoter. Two promoter variants with 9 and 8 repeats representing low activity and high activity promoter phase variants, respectively, highlighting the ability of NadR to efficiently or less efficiently repress the promoters (top panels) and NadR-independent effects on the derepressed promoter basal levels possibly due to differential contacts with the α-subunit of RNAP (bottom panels) due to different spatial organization of the NadR and RNAP contact points resulting from the different number of repeats.

In this study, we dissect the cis- and trans-acting elements as well as environmental factors that control transcriptional regulation of the *nadA* promoter in order to elucidate the mechanism by which SSR distally upstream of the P*_nadA_* promoter controls its activity. We describe a complex promoter architecture in which spontaneous changes in the number of simple sequence repeats in a tract between the most distal regulatory regions and the core promoter can alter the promoter activity and lead to phase variable expression. We have shown that the NadR repressor is the major contributor to the phase variable expression levels of the promoter as it binds to two high affinity operators flanking the SSR. One operator overlaps the −10 region of the promoter and the transcriptional start site and therefore binding of NadR is consistent with its function as a repressor through sterically hindering RNAP access to the promoter. The other high affinity operator is on the distal upstream side of the phase variable repeat in a cis-acting region that we call the GPR, which is functionally active in repressing the promoter despite its distal location (Figure 3). We have identified a single IHF binding site that is located between these two high affinity operators and we show that IHF binding to this promoter is necessary for efficient NadR-mediated repression of P*_nadA_*. The IHF binding site comprises some sequence upstream of the TAAA as well as part of the TAAA tract itself. We found that if the TAAA tract is removed, the protein no longer binds to the promoter (Figure 2) but that IHF binding is unaffected by the number of TAAA repeats. However the number of repeats changes the spacing of the DNA on the upstream and downstream flanking regions of the tract and, therefore, may influence the localisation, and possibly the orientation of proteins that bind to the operators. The ability of IHF to bend DNA may facilitate the looping of the DNA of the *nadA* promoter and bring the GPR element proximal to the core promoter elements. A looping mechanism would explain the function of such a distal operator in repression of transcription, possibly through interactions of dimers present on spatially proximal operators which lock the promoter to RNAP similar to the mechanism described for the *lac* operon [36],[37]. However, in the completely derepressed form there are still significant, albeit marginal differences in the promoter strength of variants with different numbers of repeats. The alpha-subunit of RNAP binds to the distal GPR regulatory region and also immediately upstream of the core promoter overlapping the TAAA tract which may function as UP-like elements. We propose a model in which differential distancing between the NadR operators and the contact points of RNAP result in optimal or suboptimal configuration of the protein complexes and, therefore, result in more or less efficient repression mediated by NadR and/or more or less cis-enhancement of RNAP activity on the basal promoter strength (Figure 7). Interestingly, in the Pu promoter of *Pseudomonas putida* two distinct UP elements, located close to the core promoter (−79) and distally upstream (−104), interact with RNAP α-subunits enhancing gene expression [38]. This interaction is modulated by IHF that allows the interchangeable positioning of the two α-subunits over the two UP-elements [38],[39]. This scenario resembles in part what we observe with α-subunit interaction over the *nadA* promoter.

The gonococcal homologue of NadR is FarR, which has been characterised in gonococcus as a repressor of the FarAB fatty acid resistance efflux pump [22]. FarR binds two distal operators on the *farAB* promoter (located similarly to OpI and OpII in the *nadA* promoter) and represses transcription in an IHF dependent way. It would appear from deletion analysis of the *nadA* promoter that all regulatory elements necessary for control of *nadA* expression were horizontally transferred together with the *nadA* gene, as the direct repeat delineating the border of the transferred DNA is at −193. The intriguing question is: how did the *nadA* locus, which is present in only a fraction of strains as a result of a horizontal transfer event, adopt such a complex regulatory mechanism that pre-existed in *Neisseria*. The *nadR* gene is well conserved in other *Neisseria* spp. such as *N. mucosa*, *N. cinerea, N. lactamica, N. subflavia* and *N. flavescens* and, therefore, must respond to signals in the ecological niches of all these species.

The NadR protein is a member of the MarR ligand-responsive transcriptional regulators and the majority of MarR family members are regulated by the non-covalent binding of low molecular weight ligands. These signalling molecules regulate the activity of the regulators. In this study, we have identified a putative ligand, 4HPA, which is able to relieve the DNA binding activity of NadR, thus derepressing or inducing NadA expression. This molecule is a catabolite of aromatic amino acids and it is secreted in human saliva [40] (and also urine), suggesting that the inducer is present in the oropharynx and NadA may be induced in the mucosal niche which is bathed in saliva.

The 4HPA molecule is a catabolite of the aromatic amino acids, tyrosine and phenylalanine. Two groups of bacteria, the soil inhabitants such as *P. putida* and the enteric bacteria such as *E. coli* contain pathways for the breakdown of these amino acids to succinate and pyruvate [41],[42]. However, such a pathway is not present in the meningococcal genome [43]. Nevertheless, *nadR* is present in an operon-like organization with two downstream genes, one of which shows significant homology to the HpaC small subunit of a hydroxylase involved in the conversion of 4HPA to a less toxic form (3,4-dihydroxyphenylacetic acid). It is unclear whether this operon may be involved in the utilisation of the 4HPA inducer in some way in the meningococcus, or whether it is the remnant of a partial catabolic pathway that was acquired horizontally and the 4HPA molecule simply acts as a signal inducing the expression of the adhesin, which is necessary for colonization and invasion of the mucosa.

Phase variation functions as an adaptive strategy generating spontaneous diverse sub-populations of the bacterium which may be beneficial in adapting to different microenvironments within the human host during the course of a natural infection. However, in the case of *nadA* gene regulation, this type of mechanism is bi-functional, in that the major mediation of phase variable expression levels of NadA is through repression by NadR in the absence of the correct inducer signal. Subpopulations expressing low levels of NadA through promoter phase variations still have the potential to respond to the correct niche signal, such as 4HPA, and express high levels under certain environmental or niche-specific conditions. Such variation will have an impact on the interaction with the host tissues, as well as escaping immune responses. Simple sequence repeats have been identified in distal promoter regions of known or potential virulence factors in other pathogens including *Helicobacter pylori* [44], Campylobacter jejuni [45],[46], pathogenic Burkholderia [47], *Haemophilus influenza* [32],[48], *Moraxella catarrhalis* [49], Group B *Streptococcus* [34], and pathogenic *Mycoplasma* [35], some of which have been shown to control phase variable expression through unknown mechanisms. This suggests that complex regulatory mechanisms such as what we have elucidated for NadA involving stochastic variations and environmentally-responsive transcription factors may be widely used by pathogens. Elucidating these mechanisms is important for our understanding of the intimate and complex relationship between the host and disease-causing organisms.

## Materials and Methods

### Ethics statement

All animal experiments were performed in accordance to European (directive 86/609) and Italian (D.Lgs 116/92) guidelines, regarding the protection of animals used for experimental and other scientific purposes. Such experiments were carried out under the supervision of Internal Animal Ethical Committee (AEC), Novartis Vaccine and Diagnostics. Detailed information about the policy and responsibilities can be found on the Novartis web page: (http://www.corporatecitizenship.novartis.com/business-conduct/responsible-rd/animal-welfare/our-policy.shtml). Studies are carried out by individuals who are trained and qualified in science and in the proper care, handling and use of animals and experienced with the species being studied.

### Bacterial strains and culture conditions

The *N. meningitidis* strains used in this study (Table 1) were routinely cultured, stocked, or transformed as previously described [50]. Liquid cultures were grown in GC broth (Difco) supplemented with Kellogg's supplement I, 12.5 µM Fe(NO_3_)_3_ or Mueller Hinton (MH) (Sigma, St. Louis, MO) containing 0.25% glucose and, when required, erythromycin and/or chloramphenicol were added to a final concentration of 5 µg/ml. *E. coli* DH5-α [51] and BL21(DE3) [52] cultures were grown in Luria–Bertani medium, and when required, ampicillin and/or chloramphenicol were added at 100 and 20 µg/ml, respectively.

**Table 1:** Strains and plasmids used in this study.

Name	Relevant characteristics	Reference or source
***Neisseria meningitidis***		
MC58	Clinical isolate, sequenced strain containing 9 TAAA tetranucleotide repeats in the *nadA* promoter	[43]
5/99	Clinical isolate containing 8 TAAA tetranucleotide repeats in the *nadA* promoter	Norwegian clinical isolate
BZ83	Clinical isolate containing 5 TAAA tetranucleotide repeats in the *nadA* promoter	[3]
ISS838	Clinical isolate containing 6 TAAA tetranucleotide repeats in the *nadA* promoter	[3]
961–5945	Clinical isolate containing 12 TAAA tetranucleotide repeats in the *nadA* promoter	[3]
MC-Δ1843	NadR null mutant in MC58 strain, Cm^R^	This study
5/99-Δ1843	NadR null mutant in 5/99 strain, Cm^R^	This study
BZ83-Δ1843	NadR null mutant in BZ83 strain, Cm^R^	This study
ISS-Δ1843	NadR null mutant in ISS838 strain, Cm^R^	This study
961-Δ1843	NadR null mutant in 961–5945 strain, Cm^R^	This study
MC-P(1–5)	Series of 5 derivatives of MC58, containing single copy transcriptional fusion of 5′ deletions of the *nadA* promoter fused to the *gfp* gene, Ery^R^	This study
MC-PΔ	Derivative of MC58, containing single copy transcriptional fusion of a mutant variant of the *nadA* promoter, with the tetranucleotide repeats deleted, fused to the *gfp* gene, Ery^R^	This study
MC-P2(x4–x13)	Series of 10 derivatives of MC58, containing single copy transcriptional fusion of the *nadA* P2 promoter variants, containing from 4 to 13 tetranucleotide repeats, fused to the *gfp* gene, Ery^R^	This study
Δ1843-PΔ, -P2(x8, X9)	3 Derivatives of MC-Δ1843, containing single copy transcriptional fusion of the *nadA* P2 promoter with either 0, 8 or 9 tetra nucleotide repeats, fused to the *gfp* gene, Ery^R^	This study
MC-Fko	Fur null mutant of MC58	[60]
Fko-P2(x9)	Derivative of MC-Fko containing single copy transcriptional fusion of the *nadA* P2 promoter with 9 repeats, fused to the *gfp* gene, Ery^R^	This study
**Plasmids**		
pGEMT	Cloning vector, Amp^R^	Promega
pΔ1843ko::Cm	Construct for generating knockout of the NMB1843 gene, Cm^R^	This study
pGFP	Construct for insertion of *nadA* promoter variants and mutants fused to *gfp* in single copy between ORF nmb1074 and NMB1075 in the *N. meningitidis* chromosome, Amp^R^, Ery^R^	[61]
pGX-1	Derivative pSC101 containing the *gfp* gene, CmR	[62]
pGX-nad-gfp	Derivative of pGX-1 with the *nadA* promoters cloned as a AatII/NheI fragment upstream of the *gfp* gene, Cm^R^	This study
plaw2	Expression vector for over-expression of α-subunit of RNAP under an IPTG-inducible promoter, Amp^R^	[63]
plaw2Δ256	Expression vector for over-expression of N-terminus of the α-subunit of RNAP from amino acids 1–256, under an IPTG-inducible promoter, Amp^R^	[64]
pHTT7f1-NHα	Vector for expression of N-terminal Histagged α-subunit of RNAP protein under an IPTG-inducible promoter, Amp^R^	[55]
pET15b	Expression vector for N-terminal Histagged proteins, Amp^R^	Invitrogen
pET15b-1843	pET15b derivative for expression of recombinant 1843 protein, Amp^R^	This study

### Construction of nadA promoter fusions

DNA manipulations were carried out routinely as described for standard laboratory methods [53]. Plasmid pGFP, consisting of a promoterless *gfp* gene and the *ermAM* erythromycin-resistance genes flanked by upstream and downstream regions for allelic replacement, was used to generate single copy promoter fusions by allelic exchange in *N. meningitidis* strains (Table 1). A series of 5′ deletion variants of the *nadA* promoter were generated by PCR amplification from the genome of MC58 using primers Nad-N1, Nad-N2, Nad-N3, Nad-N4 and Nad-N5 as the forward primers and Nad-Sp as the reverse primer (Table 2), generating P1, P2, P3, P4 and P5 promoter deletion fragments respectively, spanning from −273, −170, −108, −49, and −9, respectively, to +79 with respect to the transcriptional start site. Phase variant *nadA* promoters with different repeat numbers were amplified by PCR with the Nad-N2/Nad-Sp primer pair using genomic DNA as a template, derived from different clinical isolates. All promoter fragments generated, were then cloned as *Nsi*I-*Sph*I fragments into the pGFP plasmid and used for transformation of strain MC58, generating the MC-P1, MC-P2, MC-P3, MC-P4, and MC-P5 strains, respectively, for the 5′ deletion variants, and MC-P2x4, MC-P2x5, MC-P2x6, MC-P2x7, MC-P2x8, MC-P2x9, MC-P2x10, MC-P2x11, MC-P2x12, MC-P2x13 strains, for the phase variants (Table 1 and Figure 3A). The PΔ *nadA* promoter variant which lacks the TAAA tetranucleotide repeat region was generated by PCR amplification of regions upstream and downstream of the TAAA repeated tract using primer pairs Nad-N2/n85-50R and n85-50F/Nad-Sp (Table 2). Subsequently, in a second round of PCR, the upstream and downstream fragments were used in a self-priming PCR amplification for 5 cycles. The corresponding joined fragments were then amplified using the external primers Nad-N2/Nad-Sp, and cloned into pGFP, generating pGFP-PΔ. This plasmid was used for the transformation of MC58 generating, MC-PΔ, containing a *nadA* promoter fusion in which the TAAA repeated tract was substituted with an *Eco*RI site (Table 1 and Figure 3). Transformants were analyzed by colony PCR for verification of correct insertion of markers; *nadA* promoter regions were amplified and sequenced to verify that phase variation had not occurred during DNA manipulations.

**Table 2:** Oligonucleotides used in this study.

Name	Sequence	Site
Nad-N1	attcagatgcatGACGTCGACGTCCTCGATTACGAAGGC	NsiI
Nad-N2	attcagatgcatTAAGACACGACACCGGCAGAATTG	NsiI
Nad-N3	attcagatgcatCCGAACTACCTAACTGCAAG	NsiI
Nad-N4	attcagatgcatTTGCGACAATGTATTGTATATATG	NsiI
Nad-N5	attcagatgcatCTTTAATATGTAAACAAACTTGGTGG	NsiI
Nad-Sp	attcagcatgctacGCTCATTACCTTTGTGAGTGG	SphI
Nad-B1	attcaggatcctacGCTCATTACCTTTGTGAGTGG	BamHI
n-85/50F	CTACCTAACTGCAAGAATTcTTGCGACAATGTATTG	EcoRI
n-85/50R	CAATACATTGTCGCAAgAATTCTTGCAGTTAGGTAG	EcoRI
Bio-nad-N2	attcagatgcatTAAGACACGACACCGGCAGAATTG	NsiI
nad-Aa2	attcaggacgtcTAAGACACGACACCGGCAGAATTG	AatII
nad-Nh	attcagctagcCATGCTCATTACCTTTGTGAGTGG	NheI
1843-1	TACGTTCCGGCAGTTCGGCGG	
1843-2b	cgcatcctcgggatccGGGTAGGCATTGTTTAAGTCTCC	BamHI
1843-3b	caaatgcctacccggatccCGAGGATGCGTTGAACTCGTAATACGCCG	BamHI
1843-4	ACCGCTCTTCGGGCGACAGGCCGG	
1843-F	attcacatATGCCTACCCAATCAAAACATGCG	NdeI
1843-R	attcaggatcCGGCGTATTACGAGTTCAACGCATCCTCG	BamHI
IHF-Lex	ctagaACTGCAAGAATTAAATAAATAAATAAATAAATAAATTGCGAC	XbaI
IHF-Rex2	ctagaGTCGCAATTTATTTATTTATTTATTTATTTAATTCTTGCAGT	XbaI
Gpe-3	GAATTGGGACAACTCCAGTG	
1870-pe	GAATCAGGGCAGTGGTCAGAG	
Adk-PE	CGCGCCTAAAAGTAATGC	
gpr-R	gattagcatgcCGGCATTAATATCTGTTAATATGTGC	SphI
SR-F	TCGGAAGCCGTCCGTTCCGAACC	
SR-R	attatggatccATAAACGCCAAACCCACCGCGAAGGTGG	BamHI

Capital letters indicate *N. meningitidis* derived sequences, small letters indicate sequences added for cloning purposes, and underlined letters indicate restriction enzyme recognition sites.

### Construction of knockouts

To knockout the *nadR* (NMB1843) gene in the *Neisseria* background, the pΔ1843ko::Cm plasmid was constructed. Upstream and downstream flanking regions of the NMB1843 (*nadR*) gene were amplified by PCR with the 1843-1/1843-2b and 1843-3b/1843-4 primers, respectively. Then in a second round of PCR amplification the respective upstream and downstream fragments were used in a self-priming PCR amplification for 5 cycles, and then the corresponding united fragment was amplified using the external 1843-1/1843-4 primers. This product was cloned into the pGEM-T (Promega) vector and a chloramphenicol cassette from pDT2548 [54] was inserted into the unique *Bam*HI site, between the flanking regions, generating pΔ1843ko::Cm. The plasmid was linearised and used for transformation of the meningococcal strains to make the respective Δ1843 knockout mutants (Table 1).

### Expression and purification of the E. coli RNAP α-subunit and *N. meningitidis *NadR protein

The *nadR* (NMB1843) gene was amplified from the MC58 genome with the 1843-F/1843-R primer pair and cloned as a 448 bp *Nde*I-*Bam*HI fragment into the pET15b expression plasmid (Invitrogen), generating pET15–1843. For expression and purification of NadR and the α-subunit of RNAP, the pET15–1843 and pHTT7f1-NHα [55] plasmids were transformed into *E. coli* strain BL21(DE3), respectively, and the resulting strains were grown in 200 ml of Luria-Bertani medium to an OD_600_ of 0.5. Expression of the respective recombinant proteins containing N-terminal histidine tags was induced for 3 h by adding 1 mM isopropyl-D-thiogalactopyranoside (IPTG). The proteins were purified from the harvested cells by Ni-nitrilotriacetic acid (QIAGEN) affinity chromatography under nondenaturing conditions according to the manufacturer's instructions. The purified protein preparations were diluted to 1 µg/µl and dialyzed overnight against Binding Buffer (20 mM Tris-HCl pH 8, 50 mM KCL, 10 mM MgCl_2_, 0.01% NP40) containing 10% glycerol and then again overnight against Binding buffer containing 50% glycerol. The purity of the proteins was estimated to be >98% by SDS-PAGE. The concentration of the proteins in these preparations was determined by using the Bradford colorimetric assay (Bio-Rad), and aliquots of the proteins were stored at −80°C. To generate anti-NadR antibodies, 6-week-old female CD1 mice (Charles River Laboratories) were immunized with 20 µg of NadR protein given intraperitoneally, together with complete Freund's adjuvant in three doses (day 1, 21 and 35). Bleed-out samples were taken on day 49 and used for Western blot analysis.

### Western blot analysis

*N. meningitidis* colonies from overnight plate cultures were either resuspended in PBS until OD_600_ of 1 (Figure 5), or grown to logarithmic growth phase (OD_600_ of 0.24, ca. 1 h incubation) from a starter inoculum of OD_600_ of 0.05 (Figure 6). Sample volumes of 1–2 ml were harvested and normalised to a relative OD_600_ of 5 in 1 X SDS-PAGE loading buffer (50 mM Tris Cl pH 6.8, 2.5% SDS, 0.1% Bromophenol Blue, 10% glycerol, 5% beta-mercapto-Ethanol, 50 mM DTT). For Western blot analysis, 10 µg of each total protein sample in 1 X SDS-PAGE loading buffer was separated by SDS-PAGE, and transferred onto nitrocellulose membrane using an iBlot Dry Blotting System (Invitrogen). Filters were blocked overnight at 4*°*C by agitation in blocking solution (10% skimmed milk, 0.05% Tween-20, in PBS) and incubated with primary antibodies (anti-NadA, anti-NMB2091, or anti-NadR polyclonal sera) for 90 mins at 37°C. After washing, the membranes were incubated in peroxidase-conjugated anti-rabbit or anti-mouse immunoglobulin (Biorad) in blocking solution for 1 h at room temperature (RT) and the resulting signal was detected using the Supersignal West Pico chemiluminescent substrate (Pierce).

### Overexpression of RNAP α-subunit and nadA-gfp transcriptional fusion in *E. coli*

Plasmids pXG-nad for expression of NadA-GFP translational fusions were constructed from pXG-1 plasmid by substituting the 181 bp AatII/NheI fragment containing the PLtetO-1 promoter with *nadA* promoter variants amplified with nad-Aa2/nadNh primer pairs. *E. coli* strain DH5-α was co-transformed with pXG-nad and either with pLAW2 (overexpressing α-subunit) or pLAW2Δ256 (overexpressing the N-terminus of α-subunit) (Table 1). After liquid growth to an OD_600_ of 0.5 in presence of 1 mM IPTG for induction of α expression, GFP fluorescence was measured in 48 well plates using TECAN Infinite 200 with excitation wavelength of 460 nm and an emission of 510 nm. Experiments were performed in triplicate.

### Crude extract preparation, DNA affinity purification of GPR-binding protein and electrophoretic mobility shift assay (EMSA)

*N. meningitidis* strains were grown to mid-log growth phase in 100 ml of GC broth. Cells were harvested by centrifugation and resuspended in 10 ml of PBS, sonicated using UP50H Ultrasonic Processor (Hielscher) at maximum power (20 impulse of 0.8 sec each) for 5 times at 4°C with the cell debris being removed by centrifugation at 8,000×g for 15 min. The cleared crude extract was filtered using a 0.2 µm membrane with the filtrate stored at 4°C. Protein concentration was determined using the Bradford colorimetric assay. For purification of GPR-binding protein from MC58 crude extract, a probe corresponding to the GPR element spanning from −170 to −106 with respect to the MC58 *nadA* promoter was amplified using a 5′ biotinylated primer Bio-nadN2 (Invitrogen) and a non-biotinylated gpr-R reverse primer. Twenty-five pmoles of the fragment were incubated with 1.3 ml of crude extract (0.6 µg/µl) in the presence of 100 µg of salmon sperm DNA and 500 µg bovine serum albumin (BSA) to block non-specific interactions for 20 min at RT with gentle rotation. The mixture was then added to 2.5 mg of Dynabeads M-280 streptavidin (Invitrogen), previously washed 4 times with 250 µl PBS, and incubated for 20 min at RT with gentle rotation. The tube was then placed on a magnet for 2 min for magnetic separation of the beads and after 3 washes with 250 µl PBS, proteins bound to the biotinylated GPR were eluted in 400 µl of 1 M NaCl. The sample was then dialyzed overnight against H_2_O. All fractions were analysed for binding activity using an EMSA assay. The eluted fraction was further analyzed by MALDI TOF mass spectrometry. For gel shift experiments, a probe corresponding to the GPR element was amplified using the Nad-N2/gpr-R primer pair; probes corresponding to P5 and P2 promoter fragments were amplified as described in “***Construction of nadA promoter fusion***” section; a probe corresponding to 6 TAAA repeats was obtained by annealed primers IHF-Lex and IHF-Rex2; and a probe corresponding to an unrelated intergenic region Pcon (157 bp between NMB2073 and NMB2074 converging ORFs) was amplified using SR-F and SR-R. Two pmoles of each fragment were radioactively labeled at their 5′ ends with 30 µCi of (γ-^32^P)-ATP (6000 Ci/mmol; NEN) using 10 U of T4 polynucleotide kinase (New England Biolabs). The unincorporated radioactive nucleotides were removed using the TE-10 chromaspin columns (Clontech). For each binding reaction, 40 fmoles of labeled probe was incubated with increasing amounts of crude extract or recombinant purified NadR protein in 25 µl final volume of Gelshift Binding Buffer (25 mM Tris-HCl pH 7.5, 1 mM MgCl_2_, 10% glycerol) with 2 µg salmon sperm DNA as non-specific competitor, for 15 min at RT, and run on 6% native polyacrylamide gels buffered with 0.5 X TBE at 100 V for 80 min at 4°C. Gels were dried and exposed to autoradiographic films at −80°C and radioactivity was quantified using a phosphorimager and the Image Quant software (Molecular Dynamics).

### RNA preparation and primer extension analysis

*N. meningitidis* or *E. coli* strains were grown in liquid culture to logarithmic or stationary growth phase in 20 ml sample cultures. The cells were chilled by adding them to an equal volume of frozen growth medium and were pelleted by centrifugation at 2,000×g in a benchtop centrifuge at 4°C. RNA was extracted from the pelleted cells as previously described [56]. Primer extension was performed as previously reported [50] using 20 µg of total RNA and the Gpe3 primer. Quantification of the signals from the primer elongated product was performed using a Phosphoimager and ImageQuant software. For quantitative experiments, assays were performed from at least two independent biological replicates. Internal negative controls were performed on each RNA set quantifying the specific transcript of a gene whose expression is not altered, usually *adk* or *NMB1870*.

### DNase I footprinting

The *nadA* promoter region was amplified from genomic DNA from different clinical isolates with the appropriate number of repeats as major clone and from pGFP-PΔ plasmid for no repeat, using primers Nad-N1 and Nad-B1 and cloned as 320, 342, 346 and 354 bp (for no repeat, 6, 7, and 9 repeats respectively) *Nsi*I-*Bam*HI fragments into pGEMT (Promega). A radioactive probe for DNA footprinting of *nadA* promoters were prepared as follows: approximately 2 pmol of the different plasmids were linearized with BamHI, dephosphorylated, 5′ end labeled using 5 pmol of [γ-^32^P]-ATP with T4 polynucleotide kinase and digested with *Nsi*I. *nadA* promoter fragments labeled at one extremity were purified by preparative polyacrylamide gel electrophoresis (PAGE) as previously described [50], Protein-DNA complexes were formed in 50 µl of footprinting buffer (20 mM Tris-HCl, pH 7.9, 50 mM KCl, 10 mM MgCl2, 0.01% NP-40, 10% glycerol) containing approximately 20–40 fmol (10,000 c.p.m) of the labeled probe and 200 ng of sonicated salmon sperm DNA as the non-specific competitor and recombinant NadR protein, *N. gonorrhoeae* purified IHF protein [57], *E. coli* RNAP holoenzyme (USB) or α-subunit in final concentrations as indicated were incubated for 15 min at RT. Following the initial incubation, the samples were treated for 1 min at RT with 0.03 U of DNase I (Roche) and 5 mM CaCl_2_. The reactions were stopped and samples were purified and loaded on urea-6% polyacrylamide gels as previously described [58]. As a molecular weight marker, a G+A sequence reaction [59] was performed for each DNA probe and run in parallel to the corresponding footprinting reactions.

### MALDI TOF mass spectrometry

Proteins eluted from Dynabeads M-280 streptavidin column and dialyzed against H2O were dried with a Speed Vac. (Labconco) and solubilized with 20 µl of 5 mM ammonium bicarbonate containing 0.1% (wt/vol) of RapiGest SF surfactant (Waters), incubated 5 min at 95°C and digested with 2 µg of trypsin (Sequencing grade Promega). The reaction was allowed to proceed for 15 h at 37°C. An aliquot of the reaction was diluted 10 times with 0,1% (vol/vol) of trifluoroacetic acid, and 0.7 µl was directly spotted on a matrix PAC target (Prespotted AnchorChip 96, set for Proteomics, Bruker Daltonics). Air-dried spot was washed with 0.6 µl of a solution of 70% (vol/vol) ethanol, 0.1% (vol/vol) TFA. Peptide mass fingerprint spectra were recorded with a MALDI-TOF/TOF mass spectrometer UltraFlex (Bruker Daltonics). Ions generated by laser desorption at 337 nm (N_2_ laser) were recorded at an acceleration of 25 kV in the reflector mode. In general, about 200 single spectra were accumulated for improving the signal/noise ratio and analyzed by FlexAnalysis (version 2.4, Bruker Daltonics). External calibration was performed using standard peptides pre-spotted on the target. The data of MS were further analyzed through an in-house licensed MASCOT, version 2.2.1 (Matrixscience Ltd), running on a local server containing the protein sequence data downloaded from NCBI. The following parameters were used for database searches: monoisotopic mass accuracy, 75 pm, missed cleavages, 1, oxidation of methionine as variable modifications.
